# Supplementary material for: Variations in the Major Nutrient Composition of Dominant High-Yield Varieties (HYVs) in Parboiled and Polished Rice of Bangladesh
Source: Foods. 2023 Nov 1;12(21):3997. doi: 10.3390/foods12213997 (PMC10649321; doi:10.3390/foods12213997)
Supplement: Supplementary file 1 [file foods-12-03997-s001.zip › foods-2667714-supplementary.pdf]

Supplementary Table S1: Microwave digestion program

Step	Time (Min)	Power	Temperature (ºC)
1	15	1800W	Ramp to 200
2	15	1800W	Hold at 200
3	5	Cool down	

Supplementary Table S2: Instrument configuration and ICP parameters

**Parameter**	**Settings**
Torch	Mini Torch
Nebulizer	Concentric Type
Chamber	Cyclonic Type
Peristaltic Pump	Inside
Radio Frequency Power (kW)	1.20
Plasma Gas (L/min)	10.0
Auxiliary Gas (L/min)	0.60
Carrier Gas (L/min)	0.60
View Direction	Axial/Radial
Exposure time (s)	15

Supplementary Table S3: Rice varieties with their production yield

**Rice varieties**		**Season of cultivation and harvesting**	**Yield (ton ha^{-1})**	**Standard yield (ton ha^{-1})**
Boro varieties	BRRI dhan28	Planted in November-December and harvested in March-April	7.01	6.0
	BRRI dhan29		7.35	7.5
	BRRI dhan84		6.10	6.5
Transplanted Aman (T. Aman) varieties	BR11	Planted in March-April and harvested in November-December	4.52	5.0
	BRRI dhan49		4.57	5.5

Supplementary Table S4: Effects of parboiling and polishing on the proximate composition of different rice varieties in Bangladesh

Rice varieties	Energy (Kcal)	Moisture (%)	Protein (%)	Fat (%)	Ash (%)	Available Carbohydrate (%)	TDF (%)
**BR11**							
Unparboiled unpolished	351.458 ± 0.129^{a}	11.024 ± 0.002^{c}	10.521 ± 0.001^{b}	1.466 ± 0.012^{b}	1.339 ± 0.013^{b}	72.440 ± 0.017^{c}	3.210 ± 0.010^{b}
Unparboiled polished	347.415 ± 0.098^{b}	11.773 ± 0.008^{b}	8.930 ± 0.029^{d}	0.406 ± 0.006^{c}	0.405 ± 0.004^{d}	75.535 ± 0.075^{a}	2.950 ± 0.038^{d}
Parboiled unpolished	344.902 ± 0.014^{d}	12.394 ± 0.005^{a}	10.724 ± 0.021^{a}	1.506 ± 0.005^{a}	1.408 ± 0.004^{a}	70.259 ± 0.027^{d}	3.710 ± 0.006^{a}
Parboiled polished	346.110 ± 0.057^{c}	11.782 ± 0.010^{b}	9.764 ± 0.040^{c}	0.305 ± 0.005^{d}	0.543 ± 0.012^{c}	74.550 ± 0.081^{b}	3.057 ± 0.043^{c}
**BRRI dhan28**							
Unparboiled unpolished	356.731 ± 0.779^{a}	10.056 ± 0.143^{c}	8.327 ± 0.028^{b}	1.573 ± 0.009^{b}	1.220 ± 0.152^{a}	75.841 ± 0.149^{b}	2.983 ± 0.011^{c}
Unparboiled polished	342.219 ± 0.215^{d}	12.902 ± 0.002^{a}	6.960 ± 0.010^{d}	0.644 ± 0.028^{d}	0.590 ± 0.005^{b}	75.386 ± 0.051^{c}	3.518 ± 0.057^{b}
Parboiled unpolished	352.041 ± 0.168^{b}	10.906 ± 0.006^{b}	8.545 ± 0.021^{a}	1.695 ± 0.004^{a}	1.244 ± 0.334^{a}	73.691 ± 0.074^{d}	3.919 ± 0.015^{a}
Parboiled polished	349.816 ± 0.385^{c}	10.901 ± 0.066^{b}	7.030 ± 0.026^{c}	0.692 ± 0.002^{c}	0.717 ± 0.013^{b}	77.074 ± 0.257^{a}	3.586 ± 0.295^{ab}
**BRRI dhan29**							
Unparboiled unpolished	353.992 ± 0.037^{a}	10.263 ± 0.007^{d}	8.913 ± 0.011^{b}	1.271 ± 0.010^{b}	1.315 ± 0.010^{b}	75.212 ± 0.024^{c}	3.025 ± 0.005^{c}
Unparboiled polished	349.612 ± 0.077^{b}	10.907 ± 0.017^{c}	8.656 ± 0.047^{c}	0.395 ± 0.008^{c}	0.622 ± 0.009^{d}	76.295 ± 0.031^{a}	3.125 ± 0.024^{b}
Parboiled unpolished	349.772 ± 0.276^{b}	11.350 ± 0.001^{b}	9.241 ± 0.033^{a}	1.537 ± 0.031^{a}	1.354 ± 0.014^{a}	72.969 ± 0.026^{d}	3.548 ± 0.034^{a}
Parboiled polished	344.273 ± 0.183^{c}	12.229 ± 0.023^{a}	8.323 ± 0.024^{d}	0.309 ± 0.003^{d}	0.715 ± 0.006^{c}	75.673 ± 0.041^{b}	2.750 ± 0.044^{d}
**BRRI dhan49**							
Unparboiled unpolished	351.357 ± 0.106^{a}	11.026 ± 0.010^{a}	10.532 ± 0.010^{b}	1.463 ± 0.011^{b}	1.351 ± 0.022^{b}	72.402 ± 0.023^{c}	3.227 ± 0.030^{b}
Unparboiled polished	348.860 ± 2.298^{ab}	11.355 ± 0.544^{a}	8.934 ± 0.028^{d}	0.406 ± 0.002^{c}	0.457 ± 0.043^{d}	75.886 ± 0.601^{a}	2.962 ± 0.049^{c}
Parboiled unpolished	347.186 ± 0.189^{b}	11.736 ± 0.010^{a}	10.736 ± 0.034^{a}	1.501 ± 0.001^{a}	1.481 ± 0.064^{a}	70.822 ± 0.048^{d}	3.724 ± 0.034^{a}
Parboiled polished	347.329 ± 0.739^{b}	11.430 ± 0.359^{a}	9.717 ± 0.015^{c}	0.379 ± 0.002^{d}	0.703 ± 0.002^{c}	74.755 ± 0.177^{b}	3.017 ± 0.017^{c}
**BRRI dhan84**							
Unparboiled unpolished	353.052 ± 0.197^{b}	10.273 ± 0.017^{c}	9.238 ± 0.031^{b}	1.035 ± 0.010^{b}	1.333 ± 0.061^{a}	75.269 ± 0.058^{b}	2.852 ± 0.047^{a}
Unparboiled polished	351.254 ± 0.307^{c}	10.694 ± 0.061^{b}	8.004 ± 0.003^{c}	0.626 ± 0.005^{c}	0.659 ± 0.009^{b}	76.783 ± 0.112^{a}	3.234 ± 0.050^{a}
Parboiled unpolished	355.594 ± 1.227^{a}	10.674 ± 0.052^{b}	9.313 ± 0.010^{a}	1.775 ± 0.020^{a}	1.351 ± 0.002^{a}	74.298 ± 0.600^{c}	2.589 ± 0.566^{a}
Parboiled polished	350.024 ± 0.236^{c}	10.889 ± 0.053^{a}	8.028 ± 0.008^{c}	0.491 ± 0.001^{d}	0.708 ± 0.006^{b}	76.862 ± 0.066^{a}	3.023 ± 0.001^{a}

Values are presented as mean ± standard deviation of triplicate analyses, and values with the same superscript letter within the column do not differ significantly at $p < 0.05$ by one-way ANOVA

Supplementary Table S5: Percentage of nutrient loss due to the treatment of 10% DOM

**Nutrients**	**BR dhan11**		**BRRI dhan28**		**BRRI dhan29**		**BRRI dhan49**		**BRRI dhan84**	
	Unparboiled	Parboiled	Unparboiled	Parboiled	Unparboiled	Parboiled	Unparboiled	Parboiled	Unparboiled	Parboiled
Protein	-15.08	-8.92	-16.41	-17.75	-2.92	-9.92	-15.16	-9.47	-13.35	-13.78
Fat	-72.50	-79.87	-59.02	-59.25	-68.77	-79.87	-71.98	-74.67	-39.74	-72.42
Carbohydrate	4.27	6.10	-0.61	4.59	1.44	3.70	4.82	5.55	2.01	3.45
Fiber	-8.00	-17.54	17.99	-8.43	3.31	-22.46	-8.06	-18.98	13.32	16.73
Ash	-69.83	-61.70	-51.91	-42.51	-53.03	-47.29	-66.26	-52.58	-50.87	-47.41
Thiamin	-7.02	-7.29	-21.23	-16.96	-5.92	-6.86	-16.39	-12.94	-15.19	-8.73
Riboflavin	-6.90	2.70	-43.75	-39.13	3.80	-3.90	-2.86	-12.50	-6.47	4.76
Niacin	-7.29	-7.05	-25.42	-20.91	-23.07	-9.65	-2.27	-6.23	-10.64	-9.72
Pyridoxine	-13.43	-23.08	-20.90	-37.50	-47.14	-52.94	-15.85	-2.99	-22.22	-18.68
Folate	-16.70	-7.63	-55.53	-50.03	-30.25	-41.25	-0.39	-17.58	-11.45	-12.18
P	-61.80	-48.18	-2.48	-1.44	-1.70	-1.20	-0.33	-0.25	-3.91	-4.04
Zn	-10.31	-3.96	-0.44	-0.52	-1.08	-0.42	0.36	0.59	-3.27	-3.53
Ca	-1.18	3.08	-3.82	2.89	-1.84	-3.36	1.87	-0.08	-22.44	-13.05
Mg	-22.54	-21.30	0.12	1.22	-4.76	-5.66	-0.13	0.70	-14.99	-13.84
Fe	-7.37	3.43	-7.30	-4.92	-2.81	-3.37	-1.10	-0.59	-3.61	-1.40
Na	3.09	-1.70	-4.07	-1.35	3.69	-0.24	-2.45	2.00	2.68	1.40
K	-1.77	0.39	-1.57	-2.63	-0.23	-0.42	-1.19	-1.75	-0.11	0.34

Supplementary Table S6: Percentage of nutrient loss due to the treatment of parboiling

Nutrients	BR11		BRRI dhan28		BRRI dhan29		BRRI dhan49		BRRI dhan84	
	Brown	Polished	Brown	Polished	Brown	Polished	Brown	Polished	Brown	Polished
Protein	1.90	9.29	2.64	1.01	3.66	-3.81	1.93	8.77	0.83	0.33
Fat	2.73	-24.79	7.86	7.25	21.26	-21.85	2.51	-7.32	70.83	-21.81
Carbohydrate	-3.01	-1.31	-2.83	2.24	-2.98	-0.81	-2.18	-1.49	-1.29	0.10
Fiber	15.47	3.50	31.28	1.89	17.31	-11.95	15.39	1.69	-9.23	-6.49
Ash	5.49	33.88	2.19	22.16	2.53	15.05	9.61	54.01	1.00	8.12
Thiamin	-6.80	-7.08	-5.42	-0.30	-7.24	-8.16	-10.31	-6.61	-9.22	-2.30
Riboflavin	-14.94	-6.17	7.81	16.67	-2.53	-9.76	6.67	-3.92	-9.35	1.54
Niacin	0.42	0.68	-1.86	4.08	-11.53	3.90	3.29	-0.88	-3.84	-2.85
Pyridoxine	-2.99	-13.79	-4.48	-24.53	-2.86	-13.51	-18.29	-5.80	-8.08	-3.90
Folate	-9.43	0.43	3.65	16.47	6.00	-10.73	12.53	-6.88	-8.36	-9.12
P	0.22	35.94	-1.18	-0.12	-0.35	0.16	-1.21	-1.14	-0.66	-0.79
Zn	-5.36	1.18	-1.33	-1.40	-1.92	-1.26	-4.08	-3.85	1.13	0.86
Ca	-7.62	-3.65	-5.07	1.55	-0.47	-2.01	-2.02	-3.89	-10.60	0.23
Mg	-4.06	-2.54	-0.52	0.57	-0.62	-1.57	-0.89	-0.06	-2.48	-1.15
Fe	-8.00	2.90	-2.69	-0.19	-1.96	-2.52	-0.90	-0.39	-1.81	0.44
Na	3.96	-0.83	-4.45	-1.75	1.07	-2.77	-2.67	1.77	-1.40	-2.63
K	-1.05	1.13	1.02	-0.06	-0.13	-0.33	-1.10	-1.67	0.28	0.73

Supplementary Table S7: Effects of parboiling and polishing on the mineral contents of different rice varieties in Bangladesh

Rice variety	Ca (mg/100g)	Mg (mg/100g)	Fe (mg/100g)	Zn (mg/100g)	Na (mg/100g)	K (mg/100g)	P (mg/100g)
**BR11**							
Unparboiled unpolished	40.360 ± 0.761^{a}	41.014 ± 0.313^{a}	0.633 ± 0.028^{a}	2.134 ± 0.007^{a}	5.077 ± 0.044^{a}	108.422 ± 1.218^{a}	297.337 ± 2.043^{a}
Unparboiled polished	39.884 ± 0.602^{ab}	31.770 ± 0.369^{c}	0.587 ± 0.014^{b}	1.916 ± 0.005^{d}	5.278 ± 0.268^{a}	107.286 ± 0.783^{a}	113.600 ± 1.442^{c}
Parboiled unpolished	37.283 ± 0.650^{c}	39.348 ± 0.734^{b}	0.582 ± 0.008^{b}	2.020 ± 0.013^{b}	5.233 ± 0.271^{a}	106.499 ± 0.782^{a}	298.033 ± 1.002^{a}
Parboiled polished	38.429 ± 0.677^{bc}	30.964 ± 0.361^{c}	0.604 ± 0.007^{ab}	1.939 ± 0.007^{c}	5.189 ± 0.070^{a}	107.699 ± 2.302^{a}	154.433 ± 0.896^{b}
**BRRI dhan28**							
Unparboiled unpolished	37.836 ± 0.285^{a}	35.611 ± 0.448^{a}	0.557 ± 0.022^{a}	1.886 ± 0.021^{a}	5.415 ± 0.177^{a}	114.515 ± 0.484^{a}	166.567 ± 1.159^{a}
Unparboiled polished	36.392 ± 0.170^{bc}	35.655 ± 0.815^{a}	0.517 ± 0.003^{b}	1.878 ± 0.013^{a}	5.194 ± 0.053^{ab}	112.729 ± 0.544^{b}	162.433 ± 1.350^{b}
Parboiled unpolished	35.916 ± 0.521^{c}	35.426 ± 0.262^{a}	0.542 ± 0.013^{ab}	1.861 ± 0.032^{a}	5.174 ± 0.064^{ab}	115.695 ± 0.582^{a}	164.600 ± 1.400^{ab}
Parboiled polished	36.956 ± 0.261^{b}	35.858 ± 1.409^{a}	0.516 ± 0.004^{b}	1.852 ± 0.022^{a}	5.104 ± 0.042^{b}	112.657 ± 0.493^{b}	162.233 ± 1.124^{b}
**BRRI dhan29**							
Unparboiled unpolished	41.317 ± 0.360^{a}	37.960 ± 0.435^{a}	0.545 ± 0.009^{a}	1.876 ± 0.010^{a}	5.007 ± 0.194^{a}	110.947 ± 0.344^{a}	172.733 ± 0.508^{a}
Unparboiled polished	40.556 ± 0.634^{ab}	36.154 ± 0.509^{b}	0.530 ± 0.007^{ab}	1.856 ± 0.011^{ab}	5.060 ± 0.056^{a}	110.696 ± 0.771^{a}	169.800 ± 0.624^{c}
Parboiled unpolished	41.122 ± 0.190^{a}	37.724 ± 0.391^{a}	0.534 ± 0.003^{a}	1.840 ± 0.024^{ab}	5.191 ± 0.059^{a}	110.804 ± 0.539^{a}	172.133 ± 1.159^{ab}
Parboiled polished	39.740± 0.483^{b}	35.588 ± 0.497^{b}	0.516 ± 0.003^{b}	1.832 ± 0.011^{b}	5.048 ± 0.058^{a}	110.336 ± 0.986^{a}	170.067 ± 1.050^{bc}
**BRRI dhan49**							
Unparboiled unpolished	32.817 ± 0.366^{a}	30.960 ± 0.145^{a}	0.517 ± 0.007^{a}	2.044 ± 0.003^{a}	5.254 ± 0.161^{a}	112.595 ± 2.243^{a}	211.833 ± 0.702^{b}
Unparboiled polished	33.430 ± 1.486^{a}	30.919 ± 0.201^{a}	0.511 ± 0.002^{a}	2.052 ± 0.006^{a}	5.126 ± 0.025^{a}	111.258 ± 1.086^{a}	211.133 ± 0.862^{b}
Parboiled unpolished	32.153 ± 0.343^{a}	30.685 ± 0.205^{a}	0.512 ± 0.010^{a}	1.961 ± 0.033^{b}	5.114 ± 0.122^{a}	111.358 ± 1.498^{a}	209.267 ± 0.603^{a}
Parboiled polished	32.128 ± 0.806^{a}	30.900 ± 0.202^{a}	0.509 ± 0.007^{a}	1.973 ± 0.021^{b}	5.216 ± 0.201^{a}	109.404 ± 0.674^{a}	208.733 ± 0.569^{a}
**BRRI dhan84**							
Unparboiled unpolished	44.720 ± 0.800^{a}	60.333 ± 0.937^{a}	0.702 ± 0.004^{a}	2.761 ± 0.014^{b}	5.215 ± 0.100^{a}	111.299 ± 0.226^{a}	279.867 ± 1.002^{a}
Unparboiled polished	34.683 ± 0.305^{c}	51.287 ± 0.258^{b}	0.676 ± 0.022^{a}	2.670 ± 0.009^{c}	5.355 ± 0.262^{a}	111.174 ± 0.345^{a}	268.933 ± 0.862^{b}
Parboiled unpolished	39.978 ± 0.133^{b}	58.839 ± 0.211^{a}	0.689 ± 0.010^{a}	2.792 ± 0.006^{a}	5.142 ± 0.023^{a}	111.606 ± 0.475^{a}	278.033 ± 1.626^{a}
Parboiled polished	34.762 ± 0.919^{c}	50.696 ± 0.885^{b}	0.679 ± 0.009^{a}	2.693 ± 0.004^{c}	5.214 ± 0.216^{a}	111.991 ± 1.045^{a}	266.800 ± 2.615^{b}

Values are presented as mean ± standard deviation of triplicate analyses, and values with the same superscript letter within the column do not differ significantly at $p < 0.05$ by one-way ANOVA

Supplementary Table S8: Effects of parboiling and polishing on the B vitamin contents of different rice varieties in Bangladesh

Rice varieties	Thiamin (mg/100g)	Riboflavin (mg/100g)	Niacin (mg/100g)	Pyridoxine (mg/100g)	Folate (mcg/100g)
**BR11**					
Unparboiled unpolished	0.152 ± 0.003[a]	0.029 ± 0.001[a]	3.153 ± 0.040[a]	0.223 ± 0.006[a]	11.240 ± 0.070[a]
Unparboiled polished	0.141 ± 0.002[b]	0.027 ± 0.003[a]	2.923 ± 0.012[b]	0.193 ± 0.016[bc]	9.363 ± 0.166[c]
Parboiled unpolished	0.142 ± 0.002[b]	0.025 ± 0.002[a]	3.167 ± 0.045[a]	0.217 ± 0.012[ab]	10.180 ± 0.121[b]
Parboiled polished	0.131 ± 0.002[c]	0.025 ± 0.002[a]	2.943 ± 0.042[b]	0.167 ± 0.006[c]	9.403 ± 0.127[c]
**BRRI dhan28**					
Unparboiled unpolished	0.141 ± 0.001[a]	0.021 ± 0.001[a]	3.947 ± 0.035[a]	0.223 ± 0.012[a]	12.150 ± 0.434[a]
Unparboiled polished	0.111 ± 0.001[c]	0.012 ± 0.001[b]	2.943 ± 0.032[c]	0.177± 0.015[b]	5.403 ± 0.234[b]
Parboiled unpolished	0.134 ± 0.004[b]	0.023 ± 0.002[a]	3.873 ± 0.015[a]	0.213 ± 0.006[a]	12.593 ± 0.559[a]
Parboiled polished	0.111 ± 0.001[c]	0.014 ± 0.001[b]	3.063 ± 0.031[b]	0.133 ± 0.006[c]	6.293 ± 0.152[b]
**BRRI dhan29**					
Unparboiled unpolished	0.152 ± 0.003[a]	0.026 ± 0.003[a]	3.670 ± 0.040[a]	0.233 ± 0.015[a]	10.337 ± 0.375a
Unparboiled polished	0.143 ± 0.004[b]	0.027 ± 0.003[a]	2.823 ± 0.015[d]	0.123 ± 0.012[b]	7.210 ± 0.100[b]
Parboiled unpolished	0.141 ± 0.001[b]	0.026 ± 0.001[a]	3.247 ± 0.038[b]	0.227 ± 0.006[a]	10.957 ± 0.276[a]
Parboiled polished	0.131 ± 0.001[c]	0.025 ± 0.001[a]	2.933 ± 0.006[c]	0.107 ± 0.006[b]	6.437 ± 0.393[b]
**BRRI dhan49**					
Unparboiled unpolished	0.257 ± 0.008[a]	0.046 ± 0.002[a]	6.420 ± 0.184[a]	0.273 ± 0.006[a]	15.267 ± 0.148[b]
Unparboiled polished	0.218 ± 0.005[c]	0.043 ± 0.002[a]	5.737 ± 0.038[c]	0.230 ± 0.001[b]	15.207 ± 0.096[b]
Parboiled unpolished	0.233 ± 0.003[b]	0.042 ± 0.002[a]	6.173 ± 0.076[b]	0.223 ± 0.006[b]	17.180 ± 0.061[a]
Parboiled polished	0.213 ± 0.003[d]	0.044 ± 0.001[a]	5.573 ± 0.040[c]	0.217 ± 0.012[b]	14.160 ± 0.036[c]
**BRRI dhan84**					
Unparboiled unpolished	0.181 ± 0.001[a]	0.035 ± 0.004[ab]	4.250 ± 0.040[a]	0.330 ± 0.010[a]	23.953 ± 0.465[a]
Unparboiled polished	0.151 ± 0.002[c]	0.034 ± 0.003[ab]	4.153 ± 0.035[b]	0.257 ± 0.100[c]	21.210 ± 0.199[b]
Parboiled unpolished	0.162 ± 0.002[b]	0.037 ± 0.004[a]	4.390 ± 0.020[a]	0.303 ± 0.012[b]	21.950 ± 0.276[b]
Parboiled polished	0.141 ± 0.002[c]	0.033 ± 0.003[b]	4.117 ± 0.031[b]	0.247 ± 0.006[c]	19.277 ± 0.117[c]

Values are presented as mean ± standard deviation of triplicate analyses, and values with the same superscript letter within the column do not differ significantly at $p < 0.05$ by one-way ANOVA

Supplementary Table S9: Percent contribution to RDA of B vitamins and minerals by the daily intake of different varieties of unparboiled unpolished, unparboiled polished, parboiled unpolished, and parboiled polished rice

Rice varieties	Thiamin	Riboflavin	Niacin	Pyridoxine	Folate	Ca	Mg	Fe	Zn
**BR11**									
Unparboiled unpolished	32.93	4.52	68.32	36.29	14.61	15.74	36.35	12.99	48.96
Unparboiled polished	30.62	4.21	63.34	31.41	12.17	15.55	28.16	12.05	43.96
Parboiled unpolished	30.69	3.85	68.61	35.21	13.23	14.54	34.88	11.95	46.33
Parboiled polished	28.45	3.95	63.77	27.08	12.22	14.99	27.45	12.4	44.48
**BRRI dhan28**									
Unparboiled unpolished	30.6	3.3	85.5	36.3	15.8	14.8	31.6	11.4	43.3
Unparboiled polished	24.1	1.9	63.8	28.7	7	14.2	31.6	10.6	43.1
Parboiled unpolished	29	3.6	83.9	34.7	16.4	14	31.4	11.1	42.7
Parboiled polished	24.1	2.2	66.4	21.7	8.2	14.4	31.8	10.6	24.5
**BRRI dhan29**									
Unparboiled unpolished	32.93	4.11	79.52	37.92	13.44	16.11	33.65	11.19	43.04
Unparboiled polished	30.98	4.26	61.17	20.04	9.37	15.82	32.05	10.87	42.57
Parboiled unpolished	30.55	4	70.34	36.83	14.24	16.04	33.44	10.97	42.21
Parboiled polished	28.46	3.85	63.56	17.33	8.37	15.5	31.54	10.6	42.04
**BRRI dhan49**									
Unparboiled unpolished	39.22	5.46	9.08	44.42	19.85	12.8	27.44	10.61	46.9
Unparboiled polished	32.79	5.3	89.99	37.38	19.77	13.04	27.41	10.5	47.07
Parboiled unpolished	35.17	5.82	95.12	36.29	22.33	12.54	27.2	10.52	44.99
Parboiled polished	30.62	5.1	89.19	35.21	18.41	12.53	27.39	10.45	45.26
**BRRI dhan84**									
Unparboiled unpolished	55.61	7.23	139.1	53.63	31.14	17.44	53.48	14.4	63.33
Unparboiled polished	47.16	6.76	124.29	41.71	27.57	13.53	45.46	13.88	61.26
Parboiled unpolished	50.48	6.55	133.76	49.29	28.54	15.59	52.15	14.14	64.05
Parboiled polished	46.08	6.86	120.76	40.08	25.06	13.56	44.54	13.94	61.79
